# Finding MEMO—Emerging Evidence for MEMO1′s Function in Development and Disease

**DOI:** 10.3390/genes11111316

**Published:** 2020-11-06

**Authors:** Michaela D. Schotanus, Eric Van Otterloo

**Affiliations:** 1 Iowa Institute for Oral Health Research, College of Dentistry, University of Iowa, Iowa City, IA 52242, USA; michaela-schotanus@uiowa.edu; 2 Department of Anatomy and Cell Biology, Carver College of Medicine, University of Iowa, Iowa City, IA 52242, USA; 3 Department of Periodontics, College of Dentistry, University of Iowa, Iowa City, IA 52242, USA

**Keywords:** MEMO1, receptor tyrosine kinase, cytoskeleton, migration, mineralization, craniofacial

## Abstract

Although conserved throughout animal kingdoms, the protein encoded by the gene Mediator of ERBB2 Driven Cell Motility 1 or *MEMO1*, has only recently come into focus. True to its namesake, MEMO1 first emerged from a proteomic screen of molecules bound to the ERBB2 receptor and was found to be necessary for efficient cell migration upon receptor activation. While initially placed within the context of breast cancer metastasis—a pathological state that has provided tremendous insight into MEMO1′s cellular roles—MEMO1′s function has since expanded to encompass additional cancer cell types, developmental processes during embryogenesis and homeostatic regulation of adult organ systems. Owing to MEMO1′s deep conservation, a variety of model organisms have been amenable to uncovering biological facets of this multipurpose protein; facets ranging from the cellular (e.g., receptor signaling, cytoskeletal regulation, redox flux) to the organismal (e.g., mineralization and mineral homeostasis, neuro/gliogenesis, vasculogenesis) level. Although these facets emerge at the intersection of numerous biological and human disease processes, how and if they are interconnected remains to be resolved. Here, we review our current understanding of this ‘enigmatic’ molecule, its role in development and disease and open questions emerging from these previous studies.

## 1. Introduction

Since its initial isolation, more than 15 years ago [1], the enigmatic oncogene Mediator of ERBB2-driven cell motility 1 (*MEMO1*) continues to surprise, owing to its seemingly ‘swiss-army knife like’ collection of functions and interactors. With homologs identifiable from archaea, to bacteria, to eukarya [2] the protein encoded by this gene likely harbors functions essential for basic cellular processes—although these functions have undoubtedly been refined or expanded amongst organisms. Sharing structural similarity to a class of bacterial enzymes [2,3], dioxygenases, has undoubtedly shaped, yet perplexed our understanding of MEMO1′s functions in the cell. Genetic and biochemical studies from an array of eukaryotes—including yeast, worms, mice and humans—have begun to pick apart these functions and their roles in organismal health and disease. While this disparate array of model systems and biological processes have been investigated, common themes are beginning to emerge. Here, we highlight and summarize our current understanding of MEMO1 function, drawing on past studies and common themes woven throughout. We begin by covering the wealth of information concerning MEMO1′s role in receptor signaling, cell migration and control of cytoskeletal dynamics (PART I). Secondarily, we highlight more recent studies which have extended the repertoire of MEMO1′s roles, including enzymatic and in vivo functions (PART II). Finally, we summarize what is known regarding MEMO1′s role in disease (PART III). Collectively, this synopsis provides an overview of the current ‘state of knowledge’ regarding the gene *MEMO1* and its encoded protein as well as identifies numerous knowledge gaps that remain to be resolved in the years to come.

## 2. PART I

### 2.1. MEMO1 in Receptor Signaling Pathways

#### 2.1.1. Receptor Tyrosine Kinase (RTK) Signaling

Receptor signaling pathways are critical for several cellular, developmental and physiological responses. A prototypical scenario includes: signal (i.e., ligand) secretion, ligand binding—to a membrane associated receptor/coreceptor complex—and a resulting intracellular response (e.g., protein phosphorylation, gene expression changes, etc.) and a cellular outcome. A subclass of receptors, harboring an intracellular kinase domain, are receptor tyrosine kinases (RTK). Upon ligand binding, RTKs dimerize and phosphorylate tyrosine residues on themselves or their dimerization partner. These phosphorylated residues often serve as ‘docking’ sites for downstream signaling cascades [4].

**Figure 1:**
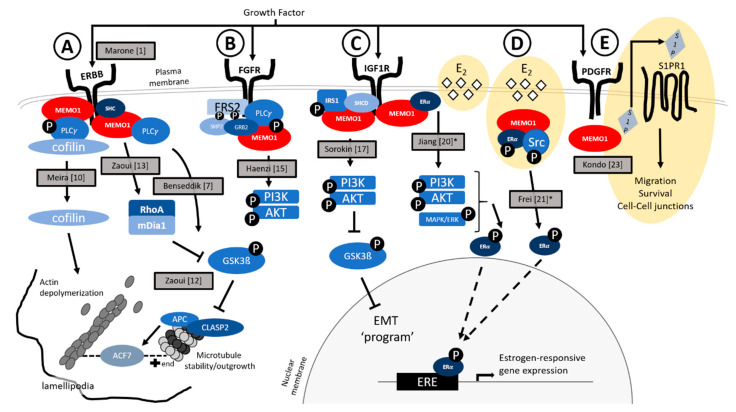
Summary of MEMO1′s role in a variety of signaling pathways, including the: (**A**) ERBB-family, (**B**) FGFR-family, (**C**) IGF1R-family, (**D**) estrogen receptor and (**E**) PDGFR and S1P/S1PR families. References are noted for each study corresponding to the depicted pathway. The pathways shaded in yellow are non-receptor tyrosine kinase signaling pathways, although it is important to note that they are intimately linked to RTK pathways. This is noted for two references in C and D by the *, which were also shown to incorporate the ERBB receptor but is not depicted here.

MEMO1 (human nomenclature used throughout) was originally discovered through its ability to bind one of these ‘docking’ sites on the erythroblastic oncogene B (ERBB2) receptor within a human epithelial breast cancer cell line [1] (Figure 1A). Belonging to the Epidermal Growth Factor Receptor (EGF/EGFR) family, the ERBB2/Human Epidermal Growth Factor Receptor 2 (HER2) receptor signaling pathway has a well-documented role in driving aggressive, metastatic breast cancers when aberrantly activated [5]. In addition to oncogenesis, the EGF/ERBB2 pathway regulates essential biological processes such as cell proliferation, adhesion, differentiation and axon guidance [6]. Through an unbiased screen, MEMO1 was shown to interact with phospho-Tyrosine (pY) 1227 on the C-terminus of ERBB2 after ligand (Heregulin, HRG) stimulation—a residue shown to be important for tumor cell migration [1]. Since its initial isolation, MEMO1′s interaction with ERBB2 is by far the most well characterized, relative to other signaling pathways [1,3,7,8,9,10,11,12,13]. Given MEMO1 does not display any obvious SH2 or phospho-tyrosine binding domains, its interaction with pY1227 was thought to be mediated, in part, through the Src homology and collagen 1 (SHC) adaptor protein [1] (Figure 1A). In support of this, MEMO1 was detected in SHC immunoprecipitates along with ERBB2 [1]. However, follow-up studies, including elucidation of the MEMO1 crystal structure, have shown that MEMO1 can bind directly to pY1227 through a binding pocket contained within the protein, originally termed a ‘vestigial active site’ [3]. While quite extensive analyses, using an array of approaches, have been used to elucidate the key residues that mediate this interaction [3,8,11] (e.g., W16, H49, Y54, H81, D189, H192, R196, R198, C244) there have been some discrepancies. For example, while residues H81, H192 and C244 were previously shown to be essential for MEMO1 binding to an ERBB2 peptide [3], follow-up studies suggested these residues may be dispensable (or less important) for MEMO1 binding. Additional structure-function and binding assays will be necessary to further clarify these interactions. Irrespective of the exact MEMO1 residues involved, MEMO1 clearly interacts with activated and thus phosphorylated, ERBB2 at the Y1227 residue. As an adapter molecule, MEMO1 was shown to link ERBB2 signaling to cell migration events (discussed in more detail below) [1]. For example, MEMO1 was shown to localize to the cell membrane of the migratory front following HRG stimulation. Further, siRNA mediated reduction of MEMO1 in a variety of cancer cell lines was shown to reduce their migratory potential, relative to controls, in response to HRG stimulation [1]. Along with ERBB2, MEMO1 was identified to complex with Phospholipase C γ (PLCγ )—an additional signaling molecule downstream of activated ERBB2—in breast cancer cells treated with HRG (Figure 1A). MEMO1 was also necessary for the phosphorylation and thus activation, of PLCγ downstream of HRG treatment—both of which were required for proper cell migration [10]. Finally, in addition to HRG, *MEMO1* knockdown cells were shown to have a reduced migratory response to additional EGFR ligands, suggesting MEMO1 likely functions downstream of additional EGF/ERBB receptors [1].

Along with the ERBB-family of RTKs, MEMO1 was also found to interact with the Fibroblast Growth Factor Receptor (FGFR) family (Figure 1B). FGF signaling has important roles in several cellular and physiological events in both the embryonic and adult organism [14]. Similar to HRG, upon Fibroblast Growth Factor 2 (FGF2) stimulation, cellular motility—of a breast cancer cell line—was reduced after siRNA-mediated knockdown of MEMO1, relative to controls [1]. In coimmunoprecipitation experiments, MEMO1 was found to associate with FGFR1 along with other proteins in the FGFR signalosome, such as the Fibroblast Growth Receptor Substrate (FRS) 1, FRS2, GRB2-Associated-Binding Protein 1 (GAB1) and Growth Factor Receptor-Bound Protein 2 (GRB2) adaptor proteins [15] (Figure 1B). While MEMO1 was not the sole mediator of signalosome assembly, it was required for optimal phosphorylation of these FGFR associated adaptor proteins. For example, upon FGF2 stimulation in *MEMO1* knockout mouse embryonic fibroblasts (MEFs) the overall phosphorylation of FRS2 was reduced and the duration of phosphorylation was markedly shorter, relative to FRS2 in control FGF2 stimulated MEFs. The net result of these changes included decreased efficiency of signalosome assembly and reduction in the activation, strength and duration of downstream signaling pathways—such as the Phosphoinositide 3-Kinase (PI3K)/Protein Kinase B (AKT)/S6K pathway (Figure 1B). In addition, FGFR binding and activation (i.e., Y783 phosphorylation) of PLCγ was impaired after ligand stimulation of *MEMO1* KO MEFs relative to controls [15] (Figure 1B). While these studies point to MEMO1 as an effector of FGFR signaling, relative to the ERBB family of RTKs, much work remains in clarifying MEMO1′s role in this pathway.

Insulin-like growth factor (IGF/IGFR) signaling is yet another RTK pathway that MEMO1 has been associated with (Figure 1C). IGF/IGFR signaling is involved in cell proliferation, differentiation and survival [16]. Like HRG and FGF2 stimulated cells, *MEMO1* knockdown cells exhibit reduced migratory capacities upon stimulation with IGF-1 [17]. Further supporting this interaction, MEMO1 and IGF1R protein expression correlated amongst various breast cancer cell lines and MEMO1 was localized to the cell membrane upon IGF-I stimulation [17]. Upon investigation of MEMO1′s role in the IGF cascade, MEMO1 was shown to have a strong binding affinity for Insulin Receptor Substrate 1 (IRS1) (Figure 1C), a signaling adaptor protein that modulates and coordinates multiple signaling events [18]. Specifically, MEMO1 was shown to bind phospho-tyrosine residues on the C-terminus of IRS1. In doing so, MEMO1 competes with SH2 domain-containing Phosphatase-2 (SHP2) for IRS1 binding, preventing SHP2-mediated IRS1 dephosphorylation. In turn, phosphorylated IRS1 activates the PI3K/AKT pathway leading to suppression of Glycogen Synthase Kinase-3 β (GSK3ß) activity and indirect activation of Nuclear Factor-kB (NFkB) activity, along with increased expression of the epithelial-mesenchymal transition (EMT) driver, Snail Family Transcriptional Repressor 1 (*SNAI1*). In support of this network, the over-expression of MEMO1 in MCF10A cells, a normal breast epithelial cell line, resulted in sustained proliferation and the formation of anchorage-independent colonies. The ability of MEMO1 to induce cell transformation was dependent on an IRS1-mediated (and SNAIL-mediated) EMT. Mesenchymal markers such as *N*-cadherin and vimentin were upregulated while epithelial markers such as *E*-cadherin, occludin and ß-catenin were downregulated in MEMO1 over-expressing cells relative to control cells [17]. Through mediating the abilities of IRS1, MEMO1-induced EMT further exhibits this protein’s capability of regulating migratory processes and metastatic potential utilizing a range of RTKs.

#### 2.1.2. Beyond RTK Signaling

While most studies have investigated MEMO1′s interaction with RTK’s, emerging evidence suggests that MEMO1 integrates with a broader range of cell signaling pathways. However, it is interesting to note that these pathways—discussed more below—coexist or interact with an RTK. These findings suggest that MEMO1 is at the intersection of these converging signaling networks.

Steroid signaling, while different from growth factor signaling, uses ligand-dependent or independent cascades to yield downstream signaling results—usually culminating in the regulation of target gene expression. Estrogen signaling has many roles in the regulation of cell growth, differentiation, angiogenesis and apoptosis through the expression of several estrogen-responsive genes [19]. While estrogen receptors (ER) are mainly found in the nucleus acting as regulators for the transcription of estrogen responsive genes, a distinct pool of extranuclear estrogen receptor (enER) is present at the plasma membrane for rapid responses that activate kinase cascades such as Mitogen-Activated Protein Kinase (MAPK) or PKB/AKT. These activated kinases can in-turn phosphorylate enER targeting it to the nucleus for gene regulation. MEMO1 has been shown to be involved in enER-mediated signaling and is found bound to enER [20,21]. One proposed mechanism for this regulation is through the already established cross talk between growth factor pathways—including IGF1R and EGFR/ERBB2—and enER signaling [22] (Figure 1C). Growth factor mediated stimulation of MAPK and PKB/AKT can promote enER nuclear localization [20]. Thus, as MEMO1 interacts with IGF1R and ERBB2, activating downstream MAPK/Extracellular Signal-Regulated Kinase (ERK)/AKT pathways, it can mediate ERα nuclear localization and function. In this regard, overexpression of MEMO1 resulted in enhanced phosphorylation of ERK/AKT and ERα, both in the presence and absence of estrogen (E_2_), while knockdown of MEMO1 showed reduced levels of phosphorylation [20]. Interestingly, MEMO1′s regulation of MAPK/AKT signaling was itself ERα-dependent, suggesting a complex interplay between these pathways. MEMO1 has also been shown to regulate the phosphorylation of ERα through mediating its interaction with c-Src kinase, a known regulator of ERα cellular localization [21] (Figure 1D). This interaction occurred downstream of both HRG and E2 stimulation, suggesting MEMO1 as a compelling link between growth factor and steroid signaling, specifically in the context of breast cancer.

Finally, in addition to RTK and steroid signaling, MEMO1 has been shown to regulate G-protein coupled receptor signaling. Traditionally, RTK signaling utilizing MEMO1 as a mediator results in the activation of ERK, AKT and PLCγ pathways. However, it was observed that despite migration defects these pathways were unchanged in *MEMO1* knock-out MEFS, relative to controls, upon Platelet-Derived Growth Factor (PDGF) α ligand stimulation [23]. These findings suggested that alternative pathways were coupled with PDGF-signaling to elicit a migratory response downstream of MEMO1 function. Sphingosine 1-phosphate Receptor 1 (S1PR1), a G-protein coupled receptor, was a strong candidate given its known role in mediating PDGF-induced migration, in part through secretion of the associated phospholipid, S1P (‘inside out’ signaling) [24] (Figure 1E). Indeed, it was observed that *MEMO1* knock-out MEFS exposed to PDGF displayed reduced extracellular S1P relative to controls. Given the importance of the S1P/S1PR signaling axis in blood vessel development, these analyses were further investigated in a human umbilical vein endothelial cell line (HUVECs) [23]. shRNA mediated knockdown of *MEMO1* in HUVECs resulted in less cell viability during serum starvation, relative to controls. These defects could be mitigated by supplying exogenous S1P, suggesting reduced extracellular S1P was responsible for the phenotypes observed upon reduction of MEMO1 [23].

Given the array of signaling pathways that MEMO1 appears to impact, and the often interconnectedness of these pathways, it will be important to identify which of these effects are direct versus indirect. Also, whether these MEMO1/receptor interactions are utilized in additional contexts, for example, during embryonic development or adult homeostasis, largely remains to be tested.

### 2.2. MEMO1 in Cell Migration and Cytoskeletal Regulation

While MEMO1 integrates with numerous signaling pathways, as discussed above, a major output of MEMO1 mediated signaling is cell migration. Cell migration is an essential event during organismal development (e.g., neural crest cells, endothelial cells, etc.), adult homeostasis (e.g., immune cells, wound healing, etc.) or during pathogenesis (e.g., tumor metastasis)—often requiring the rapid reorganization of cytoskeletal components. In vitro cell culture models have proven invaluable in deciphering the key events and components of cell migration. Often, these assays involve stimulating cells with growth factors or other ligands to initiate a signaling and thus migratory response. These assays are carried out in either non-cancerous cell lines or alternatively, cancerous cell lines, taking advantage of their already aggressive proliferative and migratory abilities. Such assays were used to identify that upon reduction or loss of MEMO1 in non-cancerous or cancerous cell lines, stimulation with a variety of growth factors and signaling molecules—including EGF, HRG, FGF, IGF, PDGF, S1P and estrogen—resulted in reduced migratory ability relative to their control counterpart (usually by 50% or more) [1,15,22,23]. Likewise, cancerous cells targeted by *MEMO1* shRNA mediated knockdown showed reduced motile abilities and invasion characteristics, relative to control cells, utilizing a variety of migratory assays [9]. It was observed that cells devoid of MEMO1 developed less invasive structures while increasing markers of polarity—such as the basal laminin V and the apical Golgi marker GM130—relative to control cells [9]. In contrast, overexpression of MEMO1 did not produce a difference in morphology or invasion in 3D culture but did produce an increased rate of migration in wound closure assays [9]. Thus, ample evidence exists linking MEMO1 function to cell migration.

How does MEMO1 execute this function downstream of cell surface receptor signaling? A variety of elegant studies have provided intriguing details into MEMO1′s regulation of these events, pinpointing cytoskeletal reorganization as a central facet in this process. The cytoskeleton, made up of microtubule and actin networks, serves a crucial role in several cellular processes such as morphogenesis, division, polarity, adhesion—as well as migration. Given the initial observations that MEMO1 functioned in cell migration the cytoskeleton was a prime suspect in mediating this effect. During HRG-induced ERBB2 activation, cells extend large cell protrusions (lamellipodia) in the direction of migration and growing microtubules invade this space to further push the cell cortex forward. siRNA mediated knockdown of *MEMO1* resulted in defects of microtubule extension into the lamellipodia, relative to control cells, following ligand stimulation [1]. Interestingly, despite altered microtubule extension, lamellipodia still appeared to form in knockdown cells, suggesting MEMO1 is involved in specific molecular and temporal events of the cell migration cascade [1]. Likewise, MEMO1—in response to ERBB2-based signaling—was found to participate only in active microtubule events (e.g., microtubule growth into lamellipodia) as the central microtubule network was unchanged in *MEMO1* knockdown cells [1].

Adding additional molecular details to these initial studies was the observation that upon HRG stimulation, MEMO1 promotes the lamellipodia membrane localization of the cytoskeletal regulators, Ras Homolog Family Member A (RhoA) and Diaphanous Related Formin 1 (mDia1) [13]. RhoA/mDia1 signaling has been studied extensively for its role in controlling several cytoskeletal processes during cell migration [25,26]. The MEMO1/RhoA/mDia1 complex, formed upon ERBB2 stimulation, was found to regulate microtubule outgrowth into the lamellipodia [12,13]. Furthermore, MEMO1/RhoA/mDia1-mediated signaling was shown to repress GSK3ß activity, resulting in the enrichment of microtubule associated proteins, Adenomatous Polyposis Coli (APC) and Cytoplasmic Linker Associated Protein 2 (CLASP2), to the cell periphery (which are known to be regulated by GSK3ß) [12]. APC and CLASP2 are essential microtubule plus-end binding proteins [27,28] that promote microtubule stability and extension into the growing lamellipodia during cell migration and have often been found to be associated with mDia1 [12,29,30]. Pushing this molecular cascade even further was the observation that the MEMO1/GSK3ß/APC-mediated network culminated in the recruitment of Actin Cross-Linking Factor 7 (ACF7) to the plasma membrane [12]. Thus, upon proper localization of APC at the cell periphery, ACF7 can assist in capturing and stabilizing the plus-end of microtubules. Interestingly, the PLCγ pathway—also implicated in ERBB2 signaling—was similarly required for ACF7 association to the cell’s leading edge and was proposed to converge with the aforementioned MEMO1/RhoA/mDia1 cascade [7], potentially in part through MEMO1′s regulation of PLCγ’s phosphorylation [10]. Adding further support to the hierarchy of these events, was the finding that targeting RhoA [13], mDia1 [12] or ACF7 [7,12] to the plasma membrane was often sufficient to overcome cellular defects observed after reduction or loss of MEMO1. Thus, loss of MEMO1 results in a disintegration of this molecular cascade during ligand-induced migration and disrupted microtubule dynamics within the lamellipodia.

Microtubules, while shown to be essential, do not operate within a vacuum during the migration processes—actin dynamics, often coupled to the microtubule network, must also be tightly controlled to sustain migratory abilities. Indeed, several of the proteins MEMO1 was shown to interact with (e.g., RhoA, mDia1, ACF7) are known ‘linkers’ of the microtubule and actin networks [26,31,32]. Ultimately, remodeling of the actin network at the cell periphery provides the physical forward force needed during cell motility [33]. Interestingly, initial observations of MEMO1′s involvement in actin dynamics stemmed from the assessment of an additional structure, focal adhesions—structures known to be disassembled by microtubules while also being coupled with the actin network. Given the microtubule defects observed in MEMO1 deficient cells, adhesion sites were examined during cell migration, revealing an increase in long-lived focal adhesions at the expense of short-lived adhesion sites—relative to control cells. Further, loss of MEMO1 was associated with a thinner α-actinin labeled lamellipodia. α-actinin is responsible for the crosslinking of F-actin, the binding of F-actin to the plasma membrane and has been shown to initiate adhesion site formation [34]. Loss of MEMO1 did not appear to influence the association/dissociation rate of α-actinin with the actin network but rather disrupted crosslinking within the actin cortex [13]—a process important for cell surface tension and cell motility [35]. These effects on the actin cortex were shown to be coupled to the previously discussed mDia/RhoA network [13]—highlighting this interconnectedness of microtubule and actin dynamics. Indeed, previous studies have found that loss of mDia1 can result in similar actin cortex defects [36]. It is interesting to note that despite defects in lamellipodial α-actinin upon loss of MEMO1, this study did not detect major changes in actin stress fibers. This contrasts with other studies in which an increase in F-actin stress fibers was observed upon reduction of MEMO1, relative to control cells [1,10]—suggesting MEMO1′s role in the actin network may be context specific.

Further integrating MEMO1 within the actin network was an unbiased screen identifying the actin binding protein cofilin-1 as a binding partner of MEMO1 [10]. When localized to the leading-edge during cell migration, cofilin remodels the actin network by depolymerizing actin filaments into the monomers required for sustained branching and growth of the F-actin network. Both MEMO1 and PLCγ were identified to facilitate ERBB2-mediated recruitment of cofilin to the lamellipodia and a complex containing ERBB2/MEMO1/ PLCγ/Cofilin could be detected following ligand induced stimulation. Intriguingly, it was also shown that MEMO1 may enhance cofilin’s actin depolymerization activity in addition to its role in recruiting cofilin to the lamellipodia. Collectively, these events were shown to be necessary for sustained cell migration downstream of HRG activation of ERBB2.

While most of these studies were conducted in breast cancer cell lines, more recent evidence suggests MEMO1′s regulation of actin and microtubule networks likely extends into other systems. For example, expression of several actin-related factors were mis-regulated upon MEMO1 knockdown in colorectal cancer (CRC) cell lines, including down-regulation of Actin Related Protein 2 (ARP2)/Neural Wiskott Aldrich Syndrome Protein (N-WASP) and up-regulation of WASP Family Verprolin Homologous Protein (WAVE-1), relative to control cells [37]. ARP2 and N-WASP work together to nucleate branching actin filaments needed to push the cell membrane forward during cell migration. Furthermore, MEMO1′s control of the cytoskeleton was recently extended into the in vivo regulation of brain cortical development. Conditional loss of *MEMO1* within specific glial cells (radial glial cells) of mice resulted in their aberrant branching during neurogenesis [38]. These defects were the result of dysregulated microtubule dynamics within the radial glial cells, ultimately resulting in disrupted brain development. Although not directly linked, it is interesting to note that CLASP2 and GSK3ß activity—known MEMO1 interactors—are also essential for proper axon growth in neurons [39,40].

In sum, substantial evidence has linked MEMO1 to cytoskeletal dynamics. While most of these interactions have been identified in breast cancer cell lines and downstream of ERBB2 signaling, it will be exciting to see whether similar networks are utilized in the context of other cell surface receptors MEMO1 interacts with. In addition, how these processes may be incorporated into other in vivo settings and developmental events remains to be seen.

## 3. PART II

### 3.1. MEMO1′s Involvement in Reactive Oxygen Species Production and the Redox Environment

MEMO1 is conserved from prokaryotes to eukaryotes (e.g., yeast, humans) [2,3], highlighting its likely importance in basic biological systems. Soon after its initial isolation, it was appreciated that MEMO1 has a structure homologous to a class of bacterial enzymes, the non-heme iron-dependent dioxygenases [3]. Indeed, more recent studies have placed MEMO1 within the context of a larger protocatechuate dioxygenase protein ‘superfamily’—of which 15 protein families are identifiable [2]. These protein families appear to have emerged from a specific protein fold (the nucleoside phosphorylase/hydrolase-peptide/amido hydrolase fold), adding additional details to MEMO1′s evolutionary history [2]. Dioxygenases are involved in aromatic ring degradation pathways, utilizing oxygen and a metal ion, within an ‘active site’ of the protein, to catalyze the opening of aromatic structures (i.e., oxidative cleavage) [2,3]. While it was speculated that RTK binding was likely a novel, derived function of the protein—after all, bacteria and even simple eukaryotes do not harbor RTKs—the question remained: Does MEMO1 still retain enzymatic and metal binding capacity? And, if so, do these functions contribute to MEMO1′s already described role in cell migration or serve some other purpose within the cell? Initial observations suggested MEMO1 may have lost these capabilities, substituting residues within its ‘active site’ for ones that would facilitate phospho-tyrosine binding [3]. However, more recent work [9] has uncovered that indeed, MEMO1 still retains enzymatic and metal binding functions.

First, it was observed that MEMO1′s ‘active site’ contained an apparent metal ion binding pocket that resembled protein domains important for metal-dependent redox biology [9]. Out of eight metal ions tested, only in the presence of copper [Cu(II)] was MEMO1 capable of generating O_2_^−^—an important intermediate product of several redox reactions. When further characterizing the important residues in MEMO1′s ‘active site’ required for copper binding and redox activity, it was found that residue H192 (previously shown to be involved in ERBB2 binding [1]) was critical, as O_2_^−^ production in the presence of Cu(II) was significantly less when this residue was mutated, relative to control [9]—although the stability and folding of the mutant variant may require further investigation [11]. The proposed mechanism by which MEMO1 could produce O_2_^−^ with this metal ion was through its interactions with the importantly conserved histidine residues found in its active site. These histidine residues could act as a base, donating an electron to Cu(II) and reducing it to Cu(I), which in turn can serve as an intermediate to reduce molecular oxygen to O_2_^−^. To stay active, it was proposed that MEMO1 would need to be ‘regenerated’ through the reduction of the active site—details that require further investigation.

How might MEMO1′s enzymatic function influence the intracellular environment? In initial studies, MEMO1 activity was associated with an oxidized cellular redox status [9]. Cell redox status in MEMO1 knockdown cells was associated with changes in mRNA expression of key players in redox homeostasis. For example, both catalase and glutamate-cysteine ligase were expressed at significantly lower levels, while glutathione peroxidase was upregulated, in MEMO1 knockdown cells, relative to controls [9]—changes indicative of a more reduced cellular environment. Interestingly, loss of MEMO1 also correlated with more reduced (inactive) forms of the redox controlled RhoA and Shc (relative to control cells), proteins previously characterized to associate with MEMO1 and regulate cytoskeletal and migratory signaling [13]. Further, although global reactive oxygen species (ROS) levels were unchanged, knockdown of MEMO1 was associated with a decrease in the local production and sustainability of ROS, specifically within the lamellipodia (or cell membrane) [9]. MEMO1 was also shown to colocalize with sites of ROS production within these cell protrusions. Finally, adding yet another level of regulation, it was found that MEMO1 was necessary for protein kinase C-mediated activation of NADPH Oxidase 1 (NOX1)—which itself produces ROS when activated. Thus, MEMO1 has been shown to contribute to the overall redox state of the cell, possibly interacting with other key redox regulators, while it creates a localized oxidized environment conducive for signaling and migratory purposes. It will be important to further dissect the causal relationships between these various molecular players and cellular events.

While the studies above were largely conducted in breast cancer cell lines, MEMO1′s integration with ROS-based mechanisms was further extended in vivo, including both in *C. elegans* and mice. It is interesting to note however, that in these settings’ loss of MEMO1 was associated with a more oxidized state—contrasting with findings in breast cancer cells. First, when *MEMO1* was knocked down by RNAi in worms, there was a significant increase in ROS within the organism, shown through a 2.5-fold increase in endogenous hydrogen peroxide levels [41]. The ROS increase was shown (both pharmacologically and genetically) to be a result of increased NADPH oxidase activity (BLI-3 in *C. elegans*), which in turn triggered a p38/MAPK-mediated activation of the transcription factor Nuclear Respiratory Factor (NRF) 1/2/3 (SKN-1 in *C. elegans*). Activated SKN-1 in turn promotes an ‘oxidative stress response’ increasing expression of several oxidative stress resistance genes. Interestingly, it was found that activation of this stress response not only promoted resistance to oxidative damage but also increased longevity in MEMO1 knockdown worms, relative to control (discussed more below). How might MEMO1 inhibit NADPH oxidase activity? Interestingly, through a screen it was identified that RHO-1, homologous to the mammalian RhoA—already known to interact with MEMO1—was required for the oxidative stress resistance found in *MEMO1* mutants. Further investigation revealed that under normal conditions, RHO-1 complexed with MEMO1, preventing RHO-1 activation of BLI-3. However, loss of MEMO1 resulted in a 2.25-fold increase in the RHO-1/BLI-3 complex, contributing to the increased ROS environment [41]. In a second study, global loss of MEMO1 within mice resulted in a more oxidized-to-reduced ratio of NAD (NAD+/NADH), specifically within serum-free bone samples [42]. While these mice showed reduced bone mineralization (discussed more below), the exact impact of an altered redox state has yet to be determined.

Collectively, these exciting findings reveal that MEMO1 still retains a metal-dependent, enzymatic function and through a variety of potential mechanisms can control the redox state of localized regions of the cell or organismal-wide. Moving forward it will be essential to determine if and the degree to which, these functions are integrated with the previously identified roles of MEMO1 in receptor-mediated signaling. Indeed, the role of ROS in cellular processes has been shown to extend into the realm of RTK signaling as locally generated ROS can potentiate signals induced by RTK activation [43]. Thus, MEMO1 could serve as a potential mediator for the integration of RTK and ROS signaling. It will also be important to determine what factors, either direct or indirect, influence the ability of MEMO1 to create a reduced or oxidized environment and how this integrates with processes of development and disease.

### 3.2. In Vivo Roles for MEMO1

While several in vitro studies have provided intricate details into the biochemical and molecular functions of MEMO1 (summarized in Table 1), new information concerning the in vivo role of MEMO1 during embryonic development and adult homeostasis continue to emerge (summarized in Table 1). These studies identify a variety of systems and processes that require MEMO1 to be executed properly. Here, we briefly highlight what is known about MEMO1 in these various settings.

#### 3.2.1. Mineralization, Skeletogenesis and Mineral Homeostasis

Mineralization is a process in the body essential for many functions. For example, vertebrates need proper mineralization to occur during both bone and tooth development—tissues requiring structural rigidity. While alterations in mineral serum levels were initially noted in *MEMO1* post-natal conditional knock-outs [15], a role for MEMO1 in skeletogenesis was revealed nearly a decade after its initial discovery through a recessive *N*-ethyl-*N*-nitrosourea (ENU) forward genetic screen in mice [45]. Mapping of the embryos presenting with craniofacial defects—including a fully penetrant cleft secondary palate and perinatal lethality—identified *MEMO1* to be the causative gene. Further evaluation of ENU mutants showed a failure of the cranial skeleton to undergo efficient mineralization. While many cranial bones were impacted, those that derived from a specific population of embryonic cells (i.e., the neural crest) appeared most affected. In addition, endochondral bones of the cranial skeleton—of neural crest origin—appeared most sensitive to loss of MEMO1. Potentially providing temporal and thus functional details, the cartilage template that precedes formation of these bones was left intact in mutants. These findings suggested that MEMO1 likely had a role in the osteogenic, rather than chondrogenic program (or the transition thereof). Expression profiling (i.e., RNA-seq) of cranial tissue from mutant and control embryos showed a significant reduction in several ossification-specific genes, including *Mmp9* and *Mmp13* and osteoblast markers, *Sp7* and *Osterix*. In contrast, genes that were upregulated were ones involved in cartilage development and the extracellular matrix. Interestingly, these changes correlated with reduced vascular invasion of the mineralizing tissue. Finally, conditional deletion of MEMO1 in the precursor cells (i.e., the neural crest) recapitulated these bone defects in the cranial skeleton—providing evidence of MEMO1′s cell autonomous role in this tissue. However, the neural crest is known to contribute to bone, cartilage and vascular support cells. Whether MEMO1 functions in one or all three cell types will require further investigation.

Additional studies revealed that MEMO1′s role in mineralization extended beyond embryogenesis and the cranial skeleton. Bones play an important role in the homeostasis and physiology of the adult organism—including the availability of essential minerals (e.g., calcium, phosphate, magnesium). A highly tuned hormone signaling axis is important for regulating the release or uptake of calcium, phosphate and other minerals stored in the bones for utilization in additional physiological processes. Initial studies investigating the postnatal roles of MEMO1 in mice, through an inducible Cre/loxP system, revealed global loss of MEMO1 resulting in a variety of defects, including overall body dysmorphology (e.g., kyphosis, ‘small stature’) correlating with altered serum calcium, vitamin D and PTH levels, relative to controls [15]. Follow up studies revealed that along with altered mineral homeostasis, bones were also significantly disturbed in MEMO1 postnatal mutants, relative to controls [42]. Bone (femur and vertebra) changes in MEMO1 mutants, relative to controls, included a reduction in trabecular volume and mineral density with a concomitant increase in cortical thickness. While both in vivo and ex vivo studies revealed no major changes to bone specific cells, (e.g., osteoblasts or osteoclasts) in mutants versus controls, defects in intracellular signaling (i.e., phosphor-ERK) were noted when mutant osteoblasts were stimulated with growth factors (EGF, FGF2) ex vivo. In addition, while the expression levels of alkaline phosphatase (ALP)—an enzyme important in bone mineralization—were unchanged, ALP activity was reduced in association with elevated NAD+/NADH in mutant bones, relative to controls. Here again, the altered bone redox state may be responsible for this decline in ALP activity, potentially through the loss of ALP dimerization potential.

While tissue specific CRE recombinases (e.g., neural crest specific knockout [45]) and ex vivo assays [42] reveal some degree of tissue/cellular autonomy for MEMO1 function during bone formation, a complex network of signaling hormones and organ systems are essential for proper mineral homeostasis. Interestingly, prompted by an overlap in phenotypes, MEMO1 was shown to facilitate FGF23 signaling in vitro [15]—which is a bone-derived signaling axis regulating phosphate/calcium homeostasis. FGF23 was upregulated in the serum of MEMO1 postnatal knockouts [15,42] (potentially a feedback mechanism in response to reduced signaling). FGF23, along with its FGF receptor and co-receptor KLOTHO, function in part, in the kidney to regulate mineral homeostasis in coordination with hormones such as calcitriol and parathyroid hormone [47,49,50,51]. However, kidney specific deletion of MEMO1 did not recapitulate the reciprocal changes seen in serum calcium or bone defects observed in ‘whole body’ postnatal MEMO1 knockouts [52]. Additionally, the regulation of *MEMO1* expression is not determined by mineral load, vitamin D or parathyroid hormone, in contrast to FGF23 or KLOTHO expression [48]. Further, administration of recombinant FGF23 in mice does not result in elevated serum calcium levels although having a profound impact on serum phosphate [53]—a mineral unaffected in MEMO1 mutants [15,42,52]. Finally, when fed a Vitamin D deficient diet, both control and MEMO1-kidney specific knock-out mice downregulated serum FGF23. In addition, several studies have now revealed the altered expression of renal and intestinal specific calcium and phosphate transport proteins in both global and kidney specific MEMO1 knockouts [15,52,54]. These findings suggest a more complex relationship in the KLOTHO/FGF23/MEMO1 signaling cascade exists and implicates MEMO1′s role in global mineral and bone homeostasis outside, or in addition to, its function in the kidney. For instance, the relative bone demineralization found in MEMO1-deficient models [42,45] could explain the combination of elevated calcemia, calciuria [42] and elevated calcium transport proteins [15,52,54]. Further, a recent report discovered increased expression of renal and intestinal magnesium channels TRPM6 and TRPM7 in ‘whole body’ postnatal and kidney-specific MEMO1 mutants. Interestingly, these changes are accompanied by an increase in serum magnesium (along with calcium) [54]. Increased serum magnesium likely prevents MEMO1 mutants from the propensity of soft tissue calcifications—a phenomenon normally observed in high serum calcium models, such as FGF23/KLOTHO mutants.

While current studies have identified many roles for MEMO1 in mineral homeostasis and skeletal development, it will be important to further clarify and connect these roles. Additional studies will help pinpoint the cell types and tissues, whether local or distant and direct or indirect, that regulate these processes. Given MEMO1′s relatively ubiquitous expression pattern it would not be surprising if a combination of tissues and systems depend on MEMO1 function to facilitate this developmental and physiological process.

#### 3.2.2. Vascular Development

One of the first defects noted upon *MEMO1* deletion in mice was the absence of homozygous mutants after birth—indicative of embryonic lethality [23]. Indeed, collecting embryos from *MEMO1*^+/−^ intercrosses revealed *MEMO1* mutants died beginning at ~embryonic day (E) 13.5. *MEMO1* mutants were identifiable from controls due to a variety of defects, including edema, pale yolk-sacs, pale embryos and hemorrhaging. Examination of mutant embryos, relative to controls, did not detect any obvious malformation in vascular organization, smooth muscle coverage or capillary bed tight junctions—suggesting at a gross level basic events of vasculo- and angiogenesis are present in mutants. However, endothelial specific (Tie2-CRE) deletion of *MEMO1* recapitulated, in ~50% of the mutants, the defects observed in *MEMO1* full knockouts—indicating some endothelial specific function for MEMO1. Interestingly, MEMO1 was shown to integrate with S1P/S1PR based signaling in vitro (discussed in ‘RTK section’ above) to regulate sprouting and cellular junction stability in an endothelial cell line—establishing concrete hypotheses that wait to be investigated in vivo. While ~50% of the *MEMO1*; Tie2-CRE knock outs recapitulated the full knock-out phenotype, the remaining ~50% survived into adulthood. What could account for these phenotypic differences? One possibility is genetic background. In fact, follow-up studies utilizing the *MEMO1* ENU mutant revealed that the early embryonic lethality, associated with vascular defects, is highly influenced by genetic background [45]. Interestingly, mutant embryos that survived into late embryonic stages (e.g., E18.5)—displaying the craniofacial bone defects described above—also displayed vascular defects within the bones analyzed. Moving forward it will be important to identify the in vivo mechanisms by which MEMO1 regulates vascular development. Although in vitro studies did not detect an interaction between MEMO1 and the Vascular Endothelial Growth Factor Receptor (VEGF/VEGFR) pathway, an overlap in craniofacial phenotypes (cleft palate, suture defects) between these mutants provides a potential link in vivo [55,56]. Indeed, the gene encoding the VEGFR ligand, *VEGFA*, was upregulated in craniofacial structures of *MEMO1* mutants [45], relative to controls, consistent with a compensating feedback loop. These findings potentially link the mineralization and vascular defects, although this relationship and the receptors involved will require further testing.

#### 3.2.3. Longevity/Aging

MEMO1 has been studied in a variety of model systems including *S. cerevisiae*, *C. elegans* and mice. While some phenotypes in *MEMO1* loss-of-function organisms likely share an underlying biochemical explanation (e.g., ROS, cellular redox state, energy homeostasis), it is difficult to draw direct links between these various eukaryotes—in part, owing to the range of complexity between a single celled organism and multicellular organisms with systemic communication between tissues. However, studies related to organismal longevity and MEMO1 have emerged, albeit with sometimes disparate outcomes.

For example, while deletion of the MEMO1 homolog in *S. cerevisiae* (*MHO1*) did not show dramatic effects in growth or survivability, a synthetic lethal screen identified MEMO1 cooperates with phosphatidylinositol phospholipase C (PLC1) to regulate cell proliferation [44]. Simultaneous loss of both MEMO1 and PLC1 in yeast limited proliferation to only ~10 generations. Interestingly, loss of MEMO1 alone did not alter actin or microtubule structures, a feature commonly affected upon loss of MEMO1 in higher eukaryotes and human MEMO1 could rescue the proliferative defects observed in MEMO1/PLC1 double mutants. These findings suggest that MEMO1 cooperates with PLC1 in regulating cell proliferation/survivability, does so likely independent of the cytoskeleton and the function responsible is retained in the human ortholog. What might this function be? One hypothesis, given PLC1′s role in mediating the oxidative stress response in yeast [57], the ability of ROS to control cell proliferation/growth [58] and the emerging evidence of MEMO1′s role in regulating the cellular redox state [9,42] is that MEMO1′s enzymatic role in ROS production is involved. However, this possibility will require further investigation.

While the molecular details of MEMO1 regulating *C. elegans* longevity, through ROS production, was discussed in more details above, it is interesting to note that loss of MEMO1, in this case, extended lifespan [41]. Owing to an indirect elevation of NADPH oxidase-mediated ROS production upon loss of MEMO1, mutant worms elicited a protective stress response, including the upregulation of oxidative stress response genes—which in turn afforded the worm an extended lifespan. Further solidifying this link was the observation that extinguishing excess ROS in MEMO1 knockdown worms reduced lifespan, while increasing NADPH oxidase expression extended lifespan. How might loss of MEMO1 result in reduced growth in yeast, while extending life in *C. elegans*? It is interesting to note that moderate levels of ROS in yeast can similarly induce a ‘protective program’ that renders the yeast more tolerant to environmental stressors and lengthens chronological lifespan [58,59,60]. However, when sufficient levels of ROS are encountered, cell growth/proliferation is slowed and at high enough levels cell death is induced [61]. It is tempting to speculate that this continuum of responses to ROS levels may underlie the distinct results observed in both systems. Alternatively, even if facets of the underlying biochemical function of MEMO1 are conserved, comparing multifactorial responses to the loss of a gene product in a multicellular organism, to a single-celled organism, may prove too complex with current information.

Interestingly, even in more complex vertebrate models, such as the mouse, an aging phenotype is reported upon loss of MEMO1 [15]. Postnatal loss of MEMO1, upon tamoxifen induced Cre/loxP-mediated whole-body deletion, results in mice exhibiting a vast array of accelerated aging phenotypes (note, opposite to that observed in *C. elegans*). For example, *MEMO1* postnatal mutants, relative to controls, display weight loss, a decrease in subcutaneous fat, small stature, kyphosis, loss of spermatozoa, graying hair, alopecia and a shortened lifespan. At a systemic level, these mice also exhibited insulin hypersensitivity, along with the previously mentioned elevated serum calcium, vitamin D and decreased PTH—again, features that mimic, to some degree, Klotho/FGF23 mutants (which themselves have an accelerated aging phenotype [62,63]). The relationship between these elements (e.g., calcium/vitamin D/PTH, Klotho/Fgf23, insulin)—regulated by endocrine systems—and longevity is well established but unravelling the cause and effect upon loss of MEMO1 and the primary organ systems directly involved will required further investigation. Also—irrespective of the disparate level of complexity between organisms (yeast, *C. elegans*, mouse)—it will be fascinating to see whether any underlying molecular processes are shared in MEMO1′s regulation of cellular to organismal ‘longevity.’

#### 3.2.4. Central Nervous System Development

Finally, another emerging system dependent on MEMO1 function during development is the brain. Initial studies, investigating differential 3′ splicing of mRNA transcripts in the cerebellum of mice, pinpointed the *MEMO1* transcript as highly susceptible to this mode of regulation [46]. Specifically, *MEMO1* was shown to switch from a short to a long 3′ untranslated region (UTR) isoform during granule cell (a key cell-type in the cerebellar cortex) differentiation. A clear question from these studies was, why the switch in isoform usage? Interestingly, the long 3′ UTR isoform was shown to provide a platform for miRNA (miR-124) targeting, resulting in the down-regulation of *MEMO1* expression as granule cells differentiated. Further, loss of MEMO1 (globally) resulted in reduced proliferation of granule cell precursors during embryogenesis, relative to controls. Collectively, these findings suggested a model in which *MEMO1′s* alternate 3′ UTR usage was a necessary step in transitioning from a proliferative granule cell precursor into a differentiated cell type. How *MEMO1* UTR usage is regulated and whether such a mechanism is deployed in additional tissues—such as osteoblasts, which were also shown to down-regulate MEMO1 transcripts during differentiation [42]—remains to be determined.

MEMO1′s involvement in brain development was further demonstrated using a tissue-specific knock-out approach in mice. Specifically, *MEMO1* deletion was targeted to radial glial progenitors (RGCs) using the *Emx1*-CRE line [38]. Deletion of *MEMO1* from this population of cells was in part, driven by the known role of cytoskeletal dynamics in RGC development, MEMO1′s known role in cytoskeletal regulation and MEMO1 mutations detected in autistic probands (discussed more below)—a disorder than can stem from RGC-related defects. Through a robust combination of mouse genetics and cell biology, this study revealed RGC morphology and migration was drastically impacted upon loss of MEMO1, including RGC hyperbranching. Given the important role of RGCs in providing a scaffold (i.e., RGC tiling) for laminar organization of neurons in the cerebral cortex, neuronal stratification was also disrupted in *MEMO1 Emx1-*CRE conditional mutants (along with other brain regions dependent on RGC tiling). What is the role of MEMO1 within the RGC’s? In depth analysis of controls and mutants revealed a major disruption of microtubule dynamics, including the alteration of key microtubule post-translational modifications, in mutants. Fittingly, vesicular trafficking, a dynamic and important part of RGC polarity—that is dependent on microtubule networks—was also disrupted in *MEMO1* conditional mutant RGC’s, relative to controls. MEMO1 was shown to associate with Calmodulin Regulated Spectrin Associated Protein Family Member 2 (CAMSAP2), a microtubule binding protein whose distribution in RGC’s was altered in *MEMO1* conditional mutants and whose over-expression could recapitulate the RGC hyperbranching phenotype observed in *MEMO1* conditional mutants. Collectively, these studies provide a detailed understanding of how MEMO1—using its ‘cytoskeletal function’—regulates brain patterning during development.

## 4. PART III

### 4.1. MEMO1 in Human Disease

#### 4.1.1. Preface: *MEMO1* as an Essential Gene

When considering the role of MEMO1 in human disease, it is important to place it within the framework of “essential genes.” Essential genes are those that are critical for cellular or organism survival (either during pre- or perinatal stages) [64,65,66,67]—with current estimates indicating that around one-third of protein coding genes are essential. Estimating how essential a gene often relies on results obtained from multiple systems including cell and animal viability screens (following gene inactivation) or human mutation burden estimates. Regarding the latter, a gene is classified as essential if a relative paucity of loss-of-function (LOF) variants exist within the gene in the general population (compared to the expected frequency under ‘neutral’ conditions)—indicating such an event is ‘intolerable’ to human life (scored as ‘probability of being LOF intolerant’) [65,66]. Using such a metric, MEMO1 was identified to be in the top 6% of all genes (~19,000 genes analyzed)—indicating LOF mutations in MEMO1 are likely detrimental to life. Interestingly, although correlative, this group of genes and their encoded proteins was also shown to have the highest mean number of protein-protein interactors and was also expressed in the broadest range of tissues [65]—features reminiscent of MEMO1. Further supporting essentiality of MEMO1 at the organismal level is the embryonic lethality associated with loss of MEMO1 in mice [23,45]. MEMO1 associated ‘embryonic lethality’ was further categorized as ‘developmental lethal’ (DL) by the International Mouse Phenotyping Consortium (IMPC) [67]. The DL category included genes that were required for life but were not categorized as ‘cellular lethal’—a term used for genes who also met a threshold for being necessary in cell viability, based on independent cell-based assays (i.e., human cell essentiality score <−0.45). It is interesting to note, however, that databases of MEMO1′s cell essentiality are often near the level of significance—with a variety of cell lines (e.g., 236 of 777 cancer cell lines, depmap portal) in which viability is dependent on MEMO1 function [64,68,69]. Identifying the contexts that make cells either dependent or independent of MEMO1 will likely reveal important insight into the protein’s function.

In sum, the ‘essentiality’ of MEMO1 is an important facet when considering the range of alterations that may be identified at the *MEMO1* locus in humans. Based on the information above, it is unlikely that very damaging mutations to MEMO1 are tolerable in the human population and may thus be highly selected against. Alternatively, single-nucleotide variants, mapped in proximity to the *MEMO1* locus display an association with anatomic traits such as male baldness or body height, which could be attributed to either or both of the associated loci and should be more closely investigated (https://www.ebi.ac.uk/gwas/genes/MEMO1). In addition to such common variants, the authors expect it is more likely that very rare de novo pathogenic variants will be identified in individuals with multisystem disorders as whole genome and whole exome sequencing become commonplace. In addition to protein coding mutations, it is also reasonable that various genomic and cellular events would lead to the mis-regulation of MEMO1 mRNA or protein expression, whether directly or indirectly. Indeed, this phenomenon has already been identified in a variety of cancer cell types, discussed more below.

#### 4.1.2. Cancer

Given the clinical observation that tumors with elevated levels of ERBB2 have a more aggressive and metastatic propensity [70] and that this phenotype is dependent on a specific phospho-tyrosine residue on ERBB2′s intracellular domain, initial studies were aimed at identifying what proteins specifically bound this residue, driving the response—enter MEMO1. Following MEMO1′s isolation from this phosphorylated residue, it quickly became apparent—utilizing breast carcinoma cell lines—that the cellular machinery required for cell migration, initiated by ERBB2 signaling, was defective when cells were depleted of MEMO1 [1]. While MEMO1′s experimental ‘history’ began with a link to cancer, new studies continue to solidify this link in a variety of new cancer contexts.

Since MEMO1′s initial isolation, numerous in vitro studies have expanded and refined our understanding of how MEMO1 regulates cell signaling and migration (discussed above) and thus, potentially in vivo tumor growth and metastasis. In vivo impacts were formally demonstrated using xenograft models, in which human breast cancer cell lines—targeted for MEMO1 knockdown—were subsequently injected into immunocompromised mice. In the first example, cells were placed in the mammary fat pad and tumor growth was monitored following estrogen treatment (a treatment shown to induce tumor growth in this model) [20]. Relative to ‘control’ tumor cells (targeted with a control shRNA), knockdown of MEMO1 resulted in absent or reduced tumor growth. In a second study, a human cell line known to spontaneously metastasize in mice, was injected and monitored following MEMO1 knockdown [9]. Relative to ‘control’ cells (targeted with a control shRNA), MEMO1 knockdown cells showed decreased metastases to the lung, associated with a decreased propensity for tumor intravasation and lung extravasation. Importantly reconstituting knockdown cells with MEMO1 (a version not targeted by the shRNA) restored levels of lung metastases to that of ‘control’ tumors. These studies provide clear evidence of MEMO1′s functional role in aspects of tumor growth and metastases, in in vivo settings.

One prediction from these studies (along with in vitro studies) was that MEMO1 expression could be altered in patient-derived tumor samples (summarized in Table 2). Indeed, upregulation of MEMO1 has now been observed in both breast [9] and colorectal cancers [37]. For example, a breast cancer tissue microarray revealed, while normal breast tissue had little to no MEMO1 protein expression, >40% of cancer tissue had increased protein expression levels [9]. These findings were consistent with a previous report identifying upregulation of *MEMO1* mRNA in ductal carcinoma in situ (i.e., preinvasive breast cancer) relative to normal breast epithelium [71]. Likewise, examination of mRNA expression levels in biopsied colorectal cancer tissue (*n* = 30), revealed ~60% of samples had elevated MEMO1 (along with HER2) expression, relative to normal colorectal tissue [37], a feature also observed at the protein level by anti-MEMO1 immunostaining. Further, although requiring validation with additional samples, MEMO1 expression levels were also found elevated (3×) in a primary pancreatic adenocarcinoma, relative to control pancreatic cells, likely as a result of *MEMO1* locus amplification (11×) [72]. In support of the biological significance of cancer-associated upregulated MEMO1 expression, were the observations that elevated MEMO1 levels correlated with a more aggressive disease prognosis and significantly reduced patient survival—in both breast [9] and colorectal cancer [37] cohorts. How might the expression of MEMO1 be upregulated in the context of cancer? While numerous mechanisms likely exist—whether direct or indirect—studies in colorectal cancer cell lines did reveal that one transcription factor complex, Aryl Hydrocarbon Receptor (AhR)/AhR Nuclear Translocator (ARNT), directly bound the MEMO1 promoter, driving MEMO1 expression in response to HER2 signaling [37]. miRNA targeting of *MEMO1* is also likely involved, as has been shown in the context of ductal carcinoma in situ [71] (also in normal embryonic development [46]). Beyond expression, protein localization also appears to be important as elevated cytoplasmic MEMO1 (as opposed to nuclear) correlated with more unfavorable parameters in breast cancer patients [9].

In yet one final example, opening novel doors to MEMO1 biology, is the recent observation that *MEMO1* circular RNA (Circ-*MEMO1*) contributes to non-small cell lung cancer (NSCLC) progression [73]. Circular RNAs are a relatively new class of RNA’s that are the result of ‘back-splicing’ of primary transcripts—resulting in highly stable (immune to nuclease digestion) molecules. In the context of Circ-*MEMO1*, back-splicing occurs between exon 5 and exon 3, generating an exon 3/4/5 circular RNA. Examination of NSCLC patients (*n* = 52) revealed that ~87% of them had elevated Circ-*MEMO1* levels in tumor tissue relative to adjacent ‘normal’ tissue. Interestingly, elevated levels of Circ-*MEMO1* were also detected within serum-derived exosomes of NSCLC patients, relative to controls. Overall, higher Circ-*MEMO1* levels correlated with advanced clinical stage, lymph node metastasis and decreased patient survival time. In vitro studies pointed to a role of Circ-*MEMO1* in binding, thus inhibiting, a miRNA (miR-101-3P), which itself inhibited KRAS—a potent mitogenic signal. Thus, increased Circ-*MEMO1* ultimately results in higher levels of KRAS, promoting cancer cell proliferation and survival. Knockdown of Circ-*MEMO1*, prior to injecting cancer cells into nude mice, resulted in smaller tumors with increased miR-101-3P levels and decreased KRAS levels, relative to control targeted cells [73]. Collectively, this study identifies yet another tumor type, albeit through a unique mechanism, that MEMO1 appears to be associated with.

In sum, while the bulk of MEMO1 knowledge has emerged from studies relevant to cancer and tumor biology, several open questions remain to be resolved. For example, is MEMO1 a ‘driver’ or ‘passenger’ gene in tumor progression? The somatic mutation frequency of the *MEMO1* locus is relatively low (intOgen [74]), suggesting that it is not a cancer driver gene in the typical sense. However, the activity of MEMO1 could still drive phenotypes relevant for malignant cell behavior (e.g., metastasis). In a similar vein, it will be important to identify the exact mechanisms by which MEMO1 expression is upregulated during tumor progression. For example, is AhR/ARNT always involved? Is upregulation a general response to over-active (i.e., oncogenic) RTK signaling or can MEMO1 be ectopically expressed without a pathogenic RTK signal? Finally, are there certain tumor subtypes (e.g., subtypes of breast, colorectal, pancreatic and lung cancer) that MEMO1 plays a more prominent role in and how might Circ-*MEMO1* RNA contribute to these various pathogenic subtypes?

#### 4.1.3. Neurodevelopmental or Neurological Disorders

As mentioned above, MEMO1 mediates proper brain morphology and organization during development—including the cerebellum [46] and cerebrum [38], among other brain regions [62]. Interestingly, neuronal disorganization is an underlying pathology found in children with autism spectrum disorder (ASD) [75]. In line with a potential MEMO1 and ASD link, *MEMO1* splice donor variants have been detected in ASD probands, including one case that introduced a premature stop codon in MEMO1 [76,77,78]. Highlighting the functional importance of this variant, it was identified that the glial-dependent neuronal defects seen in MEMO1 glial mouse mutants failed to be restored upon the reintroduction of the ASD variant, although wild-type MEMO1 could [38]. While the number of ASD probands with *MEMO1* variants, identified to date, is relatively small, copy number variation (CNV) databases such as DECIPHER [79], reveals additional individuals with deletions or duplications of regions encompassing *MEMO1*. Several of these affected individuals are reported to have a range of cognitive and neuronal impairments, including autism, intellectual disability, global developmental delay and seizures. However, given the CNVs encompass additional genes (and presumably noncoding regulatory elements), additional work would be required to definitively link alterations in MEMO1 to these pathologies.

Further extending MEMO1′s potential role in neurological pathology, MEMO1 was found to have a connection—via a combined proteomic and GWAS analysis—in the neuroinflammatory response of Parkinson’s Disease [80]. A feature that is characteristic of Parkinson’s disease is the accumulation of misfolded α-synuclein (αSyn_Agg_), in turn, eliciting a microglial inflammatory response. Using quantitative proteomics in mice, MEMO1 protein levels were significantly downregulated in microglia, following αSyn_Agg_ induced inflammation, relative to controls [80]. Further, *MEMO1* was identified, using a meta-analysis of previous Parkinson’s Disease GWAS studies, to be one of only 14 genes that was associated (suggestive association, *p* = 2.34 × 10^−5^ with Parkinson’s Disease risk and was also mis-regulated in the proteomic analysis [80]. Again, while more work is required to test the functional significance of these findings—specifically in the context of Parkinson’s Disease etiology—they suggest MEMO1′s role in neuropathology may be broader than currently appreciated.

#### 4.1.4. Additional Potential Disorders

Whether or not alterations to MEMO1 (e.g., changes in coding/noncoding sequences, expression levels, etc.) will emerge in additional developmental or adult pathologies remains to be seen. Some interesting possibilities—based on animal models and emerging human genetic information—include both hair loss and craniofacial anomalies. Regarding the former, alopecia was a reported phenotype in postnatal *MEMO1* mouse knockouts [15] and an independent mouse mutant—that also displays alopecia—had significantly altered MEMO1 expression levels in the skin [81]. In line with these observations in mice is the finding that significant GWAS SNPs, lying in or near the *MEMO1* locus, are associated with androgenous alopecia (male pattern baldness) [82,83]. Likewise, some patients in the DECIPHER [79] database with CNVs around or including *MEMO1* are reported to have sparse and thin eyebrows, sparse eyelashes, alopecia, along with additional skin abnormalities (e.g., pigmentation). Along with hair and skin pathologies, patients with *MEMO1*-containing CNVs [79] are also reported to have a variety of craniofacial anomalies, including: high arch or cleft palate, cleft lip, depressed nasal bridge/ridge, up-slanted palpebral fissures, forehead abnormalities, hyper/hypotelorism, long face, wide mouth and delayed closure of the anterior fontanelle—among other phenotypes. Again, it is critical to note that the CNVs encompass more than just the *MEMO1* locus. Thus, additional studies will be required to definitively link the causality of MEMO1 alterations to these defects. However, such findings would be congruent with the already described craniofacial defects observed in *MEMO1* mouse knockouts [45].

## 5. Conclusions

While novel roles for MEMO1 continue to emerge in a gamut of model systems, common biological themes are apparent. These include MEMO1′s role in cell migration, cytoskeletal dynamics, RTK signaling and ROS-linked biology. Likewise, in vivo experiments reveal a range of tissues in which MEMO1 function is necessary, including bone, kidney, vascular and neuronal populations—among others. Finally, MEMO1 dysregulation appears in a variety of human pathologies, most notably tumorigenesis but a link to rare developmental conditions continues to gain traction. How and if these facets of MEMO1 biology are ultimately linked or if they remain as distinct tools of the ‘swiss-army-knife’ remains to be seen.

## Figures and Tables

**Table 1:** Summary of in vitro and in vivo studies of MEMO1 function. Table reports: the main system (organism, cell line, etc.) used (A), whether RTK/receptor signaling (B), cytoskeletal (C) or reactive oxygen species (ROS) mechanisms (D) were investigated and ‘additional’ biological aspects studied (E).

A. Model System	B. RTK-Mediated Signaling and Beyond	C. Cytoskeleton	D. ROS	E. Other
In vitro cell lines	Marone 2004 [1]	Marone 2004 [1]	MacDonald 2014 [9]	
	Benseddik 2013 [7]	Benseddik 2013 [7]		
	Meira 2009 [10]	Meira 2009 [10]		
	Zaoui 2010 [12]	Zaoui 2010 [12]		
	Zaoui 2008 [13]	Zaoui 2008 [13]		
	Haenzi 2014 [15]	Bogoevska 2017 [37]		
	Sorokin 2013 [17]			
	Jiang 2013 [20]			
	Frei 2016 [21]			
	Kondo 2014 [23]			
	Bogoevska 2017 [37]			
*S. cerevisiae*	n/a	Schlatter 2012 [44]	Not studied	Viability/Longevity: Schlatter 2012 [44]
*C. elegans*	Not studied	Not studied	Ewald 2017 [41]	Longevity: Ewald 2017 [41]
*M. musculus* (Embryonic)	Not studied	Nakagawa 2019 [38]	Not studied	Viability: Kondo 2014 [23]; Van Otterloo 2016 [45]
				Vasculature: Kondo 2014 [23]; Van Otterloo 2016 [45]
				Mineralization: Van Otterloo 2016 [45]
				Neuronal Organization: Nakagawa 2019 [38]; Jereb 2018 [46]
*M. musculus* (Adult)	Not studied	Not studied	Moor 2018 [42]	Aging: Haenzi 2014 [15]
				Kidney/Serum: Haenzi 2014 [15]; Moor 2018 [42], 2016 [47], 2020 [48]
				Mineralization: Moor 2018 [42], 2020 [48]
Structure/Binding	n/a	n/a	n/a	Burroughs 2019 [2]; Qiu 2008 [3]; Feracci 2011 [8]; Newkirk 2018 [11]

**Table 2:** Summary of MEMO1 expression studies in various tumor samples. Table reports: Cancer type analyzed (A); number of samples analyzed (B); assays used to detect expression (C); basic findings of analysis (D); cellular localization for protein-based analysis along with patient outcome (E). Abbreviations: CNV, copy number variation; DCIS, ductal carcinoma in situ; IHC, immunohisto chemistry; PDAC, pancreatic ductal adenocarcinoma.

A. Type of Cancer	B. # of pt. Samples	C. Assay Utilized	D. Expression Levels	E. Cellular Location (Patient Outcome)	Ref.
Breast-mixed	407	IHC	Protein significantly higher in breast cancer compared to controls	Cytosolic and/or nuclear (cytostolic = ↑ agressiveness, ↓ prognosis)	MacDonald [9]
Colorectal	60	IHC	RNA and protein higher in colorectal cancer samples compared to controls	n/a (not reported)	Bogoevska [37]
Pre-invasive DCIS	8	(RNA) microarray	RNA signifcantly upregulated in DCIS versus patient matched control tissue	n/a (not reported)	Hannafon [71]
Pancreas-PDAC	1 (established primary cell line)	(RNA/DNA) microarray	CNV’s and RNA signifcantly increased relative to control pancreatic tissue.	n/a (died 6 weeks after pancreaticoduodenectomy)	Kalinina [72]

## Abbreviations

Accumulation of misfolded α-synuclein (αSyn_Agg_), Actin Cross-Linking Factor 7 (ACF7), Actin Related Protein 2 (ARP2), Adenomatous Polyposis Coli (APC), AhR Nuclear Translocator (ARNT), alkaline phosphatase (ALP), Aryl Hydrocarbon Receptor (AhR), autism spectrum disorder (ASD), Calmodulin Regulated Spectrin Associated Protein Family Member 2 (CAMSAP2), copy number variation (CNV), Cytoplasmic Linker Associated Protein 2 (CLASP2), developmental lethal (DL), Diaphanous Related Formin 1 (mDia1), Epidermal Growth Factor Receptor (EGF/EGFR), epithelial-mesenchymal transition (EMT), estrogen (E_2_), estrogen receptor (ER), erythroblastic oncogene B (ERBB), Extracellular Signal-Regulated Kinase (ERK), extranuclear estrogen receptor (enER), Fibroblast Growth Factor 2 (FGF2), Fibroblast Growth Factor Receptor (FGFR), Fibroblast Growth Receptor Substrate (FRS), Glycogen Synthase Kinase-3 β (GSK3ß), GRB2-Associated-Binding Protein 1 (GAB1), Growth Factor Receptor-Bound Protein 2 (GRB2), Human Epidermal Growth Factor Receptor 2 (HER2), human umbilical vein endothelial cell line (HUVECs), Insulin Receptor Substrate 1 (IRS1), Insulin-like growth factor (IGF/IGFR), International Mouse Phenotyping Consortium (IMPC), loss-of-function (LOF), Mediator of Cell Motility 1 (MEMO1), MEMO1 circular RNA (Circ-MEMO1), Mitogen-Activated Protein Kinase (MAPK), mouse embryonic fibroblasts (MEFs), NADPH Oxidase 1 (NOX1), Neural Wiskott Aldrich Syndrome Protein (N-WASP), non-small cell lung cancer (NSCLC), Nuclear Factor-kB (NFkB), Nuclear Respiratory Factor (NRF), phosphatidylinositol phospholipase C (PLC1), phospho-Tyrosine (pY), tyrosine (Y), Phosphoinositide 3-Kinase (PI3K), Phospholipase C γ (PLCγ), Platelet-Derived Growth Factor (PDGF), Protein Kinase B (AKT), radial glial progenitors (RGCs), Ras Homolog Family Member A (RhoA), reactive oxygen species (ROS), Src homology and collagen 1 (SHC), SH2 domain-containing Phosphatase-2 (SHP2), Snail Family Transcriptional Repressor 1 (*Snail1*), Sphingosine 1-phosphate Receptor 1 (S1PR1), untranslated region (UTR), Vascular Endothelial Growth Factor Receptor (VEGF/VEGFR), WASP Family Verprolin Homologous Protein (WAVE-1).

## References

[1] Marone R., Hess D., Dankort D., Muller W.J., Hynes N.E., Badache A. (2004). Memo mediates ErbB2-driven cell motility. Nat. Cell Biol..

[2] Burroughs A.M., Glasner M.E., Barry K.P., Taylor E.A., Aravind L. (2019). Oxidative opening of the aromatic ring: Tracing the natural history of a large superfamily of dioxygenase domains and their relatives. J. Biol. Chem..

[3] Qiu C., Lienhard S., Hynes N.E., Badache A., Leahy D.J. (2008). Memo is homologous to nonheme iron dioxygenases and binds an ErbB2-derived phosphopeptide in its vestigial active site. J. Biol. Chem..

[4] Lemmon M.A., Schlessinger J. (2010). Cell signaling by receptor tyrosine kinases. Cell.

[5] Hsu J.L., Hung M.C. (2016). The role of HER2, EGFR, and other receptor tyrosine kinases in breast cancer. Cancer Metastasis Rev..

[6] Wieduwilt M.J., Moasser M.M. (2008). The epidermal growth factor receptor family: Biology driving targeted therapeutics. Cell. Mol. Life Sci..

[7] Benseddik K., Sen Nkwe N., Daou P., Verdier-Pinard P., Badache A. (2013). ErbB2-dependent chemotaxis requires microtubule capture and stabilization coordinated by distinct signaling pathways. PLoS ONE.

[8] Feracci M., Pimentel C., Bornet O., Roche P., Salaun D., Badache A., Guerlesquin F. (2011). MEMO associated with an ErbB2 receptor phosphopeptide reveals a new phosphotyrosine motif. FEBS Lett..

[9] MacDonald G., Nalvarte I., Smirnova T., Vecchi M., Aceto N., Dolemeyer A., Frei A., Lienhard S., Wyckoff J., Hess D. (2014). Memo is a copper-dependent redox protein with an essential role in migration and metastasis. Sci. Signal.

[10] Meira M., Masson R., Stagljar I., Lienhard S., Maurer F., Boulay A., Hynes N.E. (2009). Memo is a cofilin-interacting protein that influences PLCgamma1 and cofilin activities, and is essential for maintaining directionality during ErbB2-induced tumor-cell migration. J. Cell Sci..

[11] Newkirk M.L., Rubenstein K.J., Kim J.Y., Labrecque C.L., Airas J., Taylor C.A., Evans H.D., McKoy Q., Parish C.A., Pollock J.A. (2018). Analysis of MEMO1 Binding Specificity for ErbB2 Using Fluorescence Polarization and Molecular Dynamics Simulations. Biochemistry.

[12] Zaoui K., Benseddik K., Daou P., Salaun D., Badache A. (2010). ErbB2 receptor controls microtubule capture by recruiting ACF7 to the plasma membrane of migrating cells. Proc. Natl. Acad. Sci. USA.

[13] Zaoui K., Honore S., Isnardon D., Braguer D., Badache A. (2008). Memo-RhoA-mDia1 signaling controls microtubules, the actin network, and adhesion site formation in migrating cells. J. Cell Biol..

[14] Mossahebi-Mohammadi M., Quan M., Zhang J.S., Li X. (2020). FGF Signaling Pathway: A Key Regulator of Stem Cell Pluripotency. Front. Cell Dev. Biol..

[15] Haenzi B., Bonny O., Masson R., Lienhard S., Dey J.H., Kuro-o M., Hynes N.E. (2014). Loss of Memo, a novel FGFR regulator, results in reduced lifespan. FASEB J..

[16] Hakuno F., Takahashi S.I. (2018). IGF1 receptor signaling pathways. J. Mol. Endocrinol..

[17] Sorokin A.V., Chen J. (2013). MEMO1, a new IRS1-interacting protein, induces epithelial-mesenchymal transition in mammary epithelial cells. Oncogene.

[18] Gorgisen G., Gulacar I.M., Ozes O.N. (2017). The role of insulin receptor substrate (IRS) proteins in oncogenic transformation. Cell. Mol. Biol. (Noisy-le-grand).

[19] Marino M., Galluzzo P., Ascenzi P. (2006). Estrogen signaling multiple pathways to impact gene transcription. Curr. Genom..

[20] Jiang K., Yang Z., Cheng L., Wang S., Ning K., Zhou L., Lin J., Zhong H., Wang L., Li Y. (2013). Mediator of ERBB2-driven cell motility (MEMO) promotes extranuclear estrogen receptor signaling involving the growth factor receptors IGF1R and ERBB2. J. Biol. Chem..

[21] Frei A., MacDonald G., Lund I., Gustafsson J.A., Hynes N.E., Nalvarte I. (2016). Memo interacts with c-Src to control Estrogen Receptor alpha sub-cellular localization. Oncotarget.

[22] Song R.X., Chen Y., Zhang Z., Bao Y., Yue W., Wang J.P., Fan P., Santen R.J. (2010). Estrogen utilization of IGF-1-R and EGF-R to signal in breast cancer cells. J. Steroid Biochem. Mol. Biol..

[23] Kondo S., Bottos A., Allegood J.C., Masson R., Maurer F.G., Genoud C., Kaeser P., Huwiler A., Murakami M., Spiegel S. (2014). Memo has a novel role in S1P signaling and is [corrected] crucial for vascular development. PLoS ONE.

[24] Rosenfeldt H.M., Hobson J.P., Maceyka M., Olivera A., Nava V.E., Milstien S., Spiegel S. (2001). EDG-1 links the PDGF receptor to Src and focal adhesion kinase activation leading to lamellipodia formation and cell migration. FASEB J..

[25] Wittmann T., Waterman-Storer C.M. (2001). Cell motility: Can Rho GTPases and microtubules point the way?. J. Cell Sci..

[26] Ishizaki T., Morishima Y., Okamoto M., Furuyashiki T., Kato T., Narumiya S. (2001). Coordination of microtubules and the actin cytoskeleton by the Rho effector mDia1. Nat. Cell Biol..

[27] Drabek K., van Ham M., Stepanova T., Draegestein K., van Horssen R., Sayas C.L., Akhmanova A., Ten Hagen T., Smits R., Fodde R. (2006). Role of CLASP2 in microtubule stabilization and the regulation of persistent motility. Curr. Biol..

[28] Aoki K., Taketo M.M. (2007). Adenomatous polyposis coli (APC): A multi-functional tumor suppressor gene. J. Cell Sci..

[29] Wen Y., Eng C.H., Schmoranzer J., Cabrera-Poch N., Morris E.J., Chen M., Wallar B.J., Alberts A.S., Gundersen G.G. (2004). EB1 and APC bind to mDia to stabilize microtubules downstream of Rho and promote cell migration. Nat. Cell Biol..

[30] Akhmanova A., Hoogenraad C.C., Drabek K., Stepanova T., Dortland B., Verkerk T., Vermeulen W., Burgering B.M., De Zeeuw C.I., Grosveld F. (2001). Clasps are CLIP-115 and -170 associating proteins involved in the regional regulation of microtubule dynamics in motile fibroblasts. Cell.

[31] Wu X., Kodama A., Fuchs E. (2008). ACF7 regulates cytoskeletal-focal adhesion dynamics and migration and has ATPase activity. Cell.

[32] Kaverina I., Straube A. (2011). Regulation of cell migration by dynamic microtubules. Semin. Cell Dev. Biol..

[33] Pollard T.D., Borisy G.G. (2003). Cellular motility driven by assembly and disassembly of actin filaments. Cell.

[34] Sjoblom B., Salmazo A., Djinovic-Carugo K. (2008). Alpha-actinin structure and regulation. Cell. Mol. Life Sci..

[35] Chugh P., Paluch E.K. (2018). The actin cortex at a glance. J. Cell Sci..

[36] Chugh P., Clark A.G., Smith M.B., Cassani D.A.D., Dierkes K., Ragab A., Roux P.P., Charras G., Salbreux G., Paluch E.K. (2017). Actin cortex architecture regulates cell surface tension. Nat. Cell Biol..

[37] Bogoevska V., Wolters-Eisfeld G., Hofmann B.T., El Gammal A.T., Mercanoglu B., Gebauer F., Vashist Y.K., Bogoevski D., Perez D., Gagliani N. (2017). HRG/HER2/HER3 signaling promotes AhR-mediated Memo-1 expression and migration in colorectal cancer. Oncogene.

[38] Nakagawa N., Plestant C., Yabuno-Nakagawa K., Li J., Lee J., Huang C.W., Lee A., Krupa O., Adhikari A., Thompson S. (2019). Memo1-Mediated Tiling of Radial Glial Cells Facilitates Cerebral Cortical Development. Neuron.

[39] Kim Y.T., Hur E.M., Snider W.D., Zhou F.Q. (2011). Role of GSK3 Signaling in Neuronal Morphogenesis. Front. Mol. Neurosci..

[40] Hur E.M., Lee B.D., Kim S.J., Xu  W.L., Zhou F.Q. (2011). GSK3 controls axon growth via CLASP-mediated regulation of growth cone microtubules. Genes Dev..

[41] Ewald C.Y., Hourihan J.M., Bland M.S., Obieglo C., Katic I., Moronetti Mazzeo L.E., Alcedo J., Blackwell T.K., Hynes N.E. (2017). NADPH oxidase-mediated redox signaling promotes oxidative stress resistance and longevity through memo-1 in C. elegans. Elife.

[42] Moor M.B., Ramakrishnan S.K., Legrand F., Dolder S., Siegrist M., Durussel F., Centeno G., Firsov D., Hynes N.E., Hofstetter W. (2018). Redox-Dependent Bone Alkaline Phosphatase Dysfunction Drives Part of the Complex Bone Phenotype in Mice Deficient for Memo1. JBMR Plus.

[43] Ostman A., Frijhoff J., Sandin A., Bohmer F.D. (2011). Regulation of protein tyrosine phosphatases by reversible oxidation. J. Biochem..

[44] Schlatter I.D., Meira M., Ueberschlag V., Hoepfner D., Movva R., Hynes N.E. (2012). MHO1, an evolutionarily conserved gene, is synthetic lethal with PLC1; Mho1p has a role in invasive growth. PLoS ONE.

[45] Van Otterloo E., Feng W., Jones K.L., Hynes N.E., Clouthier D.E., Niswander L., Williams T. (2016). MEMO1 drives cranial endochondral ossification and palatogenesis. Dev. Biol..

[46] Jereb S., Hwang H.W., Van Otterloo E., Govek E.E., Fak J.J., Yuan Y., Hatten M.E., Darnell R.B. (2018). Differential 3’ Processing of Specific Transcripts Expands Regulatory and Protein Diversity Across Neuronal Cell Types. Elife.

[47] Moor M.B., Bonny O. (2016). Ways of calcium reabsorption in the kidney. Am. J. Physiol. Renal. Physiol..

[48] Moor M.B., Bonny O. (2020). Memo1 gene expression in kidney and bone is unaffected by dietary mineral load and calciotropic hormones. Physiol. Rep..

[49] Erben R.G., Andrukhova O. (2017). FGF23-Klotho signaling axis in the kidney. Bone.

[50] Olauson H., Lindberg K., Amin R., Jia T., Wernerson A., Andersson G., Larsson T.E. (2012). Targeted deletion of Klotho in kidney distal tubule disrupts mineral metabolism. J. Am. Soc. Nephrol..

[51] Lindberg K., Amin R., Moe O.W., Hu M.C., Erben R.G., Ostman Wernerson A., Lanske B., Olauson H., Larsson T.E. (2014). The kidney is the principal organ mediating klotho effects. J. Am. Soc. Nephrol..

[52] Moor M.B., Haenzi B., Legrand F., Koesters R., Hynes N.E., Bonny O. (2018). Renal Memo1 Differentially Regulates the Expression of Vitamin D-Dependent Distal Renal Tubular Calcium Transporters. Front. Physiol..

[53] Shimada T., Mizutani S., Muto T., Yoneya T., Hino R., Takeda S., Takeuchi Y., Fujita T., Fukumoto S., Yamashita T. (2001). Cloning and characterization of FGF23 as a causative factor of tumor-induced osteomalacia. Proc. Natl. Acad. Sci. USA.

[54] Moor M.B., Ramakrishnan S.K., Legrand F., Bachtler M., Koesters R., Hynes N.E., Pasch A., Bonny O. (2020). Elevated serum magnesium lowers calcification propensity in Memo1-deficient mice. PLoS ONE.

[55] Stalmans I., Lambrechts D., De Smet F., Jansen S., Wang J., Maity S., Kneer P., von der Ohe M., Swillen A., Maes C. (2003). VEGF: A modifier of the del22q11 (DiGeorge) syndrome?. Nat. Med..

[56] Wiszniak S., Mackenzie F.E., Anderson P., Kabbara S., Ruhrberg C., Schwarz Q. (2015). Neural crest cell-derived VEGF promotes embryonic jaw extension. Proc. Natl. Acad. Sci. USA.

[57] Cooper K.F., Mallory M.J., Strich R. (1999). Oxidative stress-induced destruction of the yeast C-type cyclin Ume3p requires phosphatidylinositol-specific phospholipase C and the 26S proteasome. Mol. Cell. Biol..

[58] Schieber M., Chandel N.S. (2014). ROS function in redox signaling and oxidative stress. Curr. Biol..

[59] Pan Y., Schroeder E.A., Ocampo A., Barrientos A., Shadel G.S. (2011). Regulation of yeast chronological life span by TORC1 via adaptive mitochondrial ROS signaling. Cell Metab..

[60] Mesquita A., Weinberger M., Silva A., Sampaio-Marques B., Almeida B., Leao C., Costa V., Rodrigues F., Burhans W.C., Ludovico P. (2010). Caloric restriction or catalase inactivation extends yeast chronological lifespan by inducing H_2_O_2_ and superoxide dismutase activity. Proc. Natl. Acad. Sci. USA.

[61] Perrone G.G., Tan S.X., Dawes I.W. (2008). Reactive oxygen species and yeast apoptosis. Biochim. Biophys. Acta.

[62] Kuro-o M., Matsumura Y., Aizawa H., Kawaguchi H., Suga T., Utsugi T., Ohyama Y., Kurabayashi M., Kaname T., Kume E. (1997). Mutation of the mouse klotho gene leads to a syndrome resembling ageing. Nature.

[63] Shimada T., Kakitani M., Yamazaki Y., Hasegawa H., Takeuchi Y., Fujita T., Fukumoto S., Tomizuka K., Yamashita T. (2004). Targeted ablation of Fgf23 demonstrates an essential physiological role of FGF23 in phosphate and vitamin D metabolism. J. Clin. Investig..

[64] Blomen V.A., Majek P., Jae L.T., Bigenzahn J.W., Nieuwenhuis J., Staring J., Sacco R., van Diemen F.R., Olk N., Stukalov A. (2015). Gene essentiality and synthetic lethality in haploid human cells. Science.

[65] Karczewski K.J., Francioli L.C., Tiao G., Cummings B.B., Alfoldi J., Wang Q., Collins R.L., Laricchia K.M., Ganna A., Birnbaum D.P. (2020). The mutational constraint spectrum quantified from variation in 141,456 humans. Nature.

[66] Lek M., Karczewski K.J., Minikel E.V., Samocha K.E., Banks E., Fennell T., O’Donnell-Luria A.H., Ware J.S., Hill A.J., Cummings B.B. (2016). Analysis of protein-coding genetic variation in 60,706 humans. Nature.

[67] Cacheiro P., Munoz-Fuentes V., Murray S.A., Dickinson M.E., Bucan M., Nutter L.M.J., Peterson K.A., Haselimashhadi H., Flenniken A.M., Morgan H. (2020). Human and mouse essentiality screens as a resource for disease gene discovery. Nat. Commun..

[68] Tsherniak A., Vazquez F., Montgomery P.G., Weir B.A., Kryukov G., Cowley G.S., Gill S., Harrington W.F., Pantel S., Krill-Burger J.M. (2017). Defining a Cancer Dependency Map. Cell.

[69] Meyers R.M., Bryan J.G., McFarland J.M., Weir B.A., Sizemore A.E., Xu H., Dharia N.V., Montgomery P.G., Cowley G.S., Pantel S. (2017). Computational correction of copy number effect improves specificity of CRISPR-Cas9 essentiality screens in cancer cells. Nat. Genet..

[70] Bazley L.A., Gullick W.J. (2005). The epidermal growth factor receptor family. Endocr. Relat. Cancer.

[71] Hannafon B.N., Sebastiani P., de las Morenas A., Lu J., Rosenberg C.L. (2011). Expression of microRNA and their gene targets are dysregulated in preinvasive breast cancer. Breast Cancer Res..

[72] Kalinina T., Gungor C., Thieltges S., Moller-Krull M., Penas E.M., Wicklein D., Streichert T., Schumacher U., Kalinin V., Simon R. (2010). Establishment and characterization of a new human pancreatic adenocarcinoma cell line with high metastatic potential to the lung. BMC Cancer.

[73] Ding C., Xi G., Wang G., Cui D., Zhang B., Wang H., Jiang G., Song J., Xu G., Wang J. (2020). Exosomal Circ-MEMO1 Promotes the Progression and Aerobic Glycolysis of Non-small Cell Lung Cancer Through Targeting MiR-101-3p/KRAS Axis. Front. Genet..

[74] Martinez-Jimenez F., Muinos F., Sentis I., Deu-Pons J., Reyes-Salazar I., Arnedo-Pac C., Mularoni L., Pich O., Bonet J., Kranas H. (2020). A compendium of mutational cancer driver genes. Nat. Rev. Cancer.

[75] Stoner R., Chow M.L., Boyle M.P., Sunkin S.M., Mouton P.R., Roy S., Wynshaw-Boris A., Colamarino S.A., Lein E.S., Courchesne E. (2014). Patches of disorganization in the neocortex of children with autism. N. Engl. J. Med..

[76] De Rubeis S., He X., Goldberg A.P., Poultney C.S., Samocha K., Cicek A.E., Kou Y., Liu L., Fromer M., Walker S. (2014). Synaptic, transcriptional and chromatin genes disrupted in autism. Nature.

[77] Iossifov I., O’Roak B.J., Sanders S.J., Ronemus M., Krumm N., Levy D., Stessman H.A., Witherspoon K.T., Vives L., Patterson K.E. (2014). The contribution of de novo coding mutations to autism spectrum disorder. Nature.

[78] Nguyen H.T., Bryois J., Kim A., Dobbyn A., Huckins L.M., Munoz-Manchado A.B., Ruderfer D.M., Genovese G., Fromer M., Xu X. (2017). Integrated Bayesian analysis of rare exonic variants to identify risk genes for schizophrenia and neurodevelopmental disorders. Genome Med..

[79] Firth H.V., Richards S.M., Bevan A.P., Clayton S., Corpas M., Rajan D., Van Vooren S., Moreau Y., Pettett R.M., Carter N.P. (2009). DECIPHER: Database of Chromosomal Imbalance and Phenotype in Humans Using Ensembl Resources. Am. J. Hum. Genet..

[80] Sarkar S., Dammer E.B., Malovic E., Olsen A.L., Raza S.A., Gao T., Xiao H., Oliver D.L., Duong D., Joers V. (2020). Molecular Signatures of Neuroinflammation Induced by alphaSynuclein Aggregates in Microglial Cells. Front. Immunol..

[81] Liu B., Xu Y., Li W.L., Zeng L. (2015). Proteomic analysis of differentially expressed skin proteins in iRhom2(Uncv) mice. BMB Rep..

[82] Hagenaars S.P., Hill W.D., Harris S.E., Ritchie S.J., Davies G., Liewald D.C., Gale C.R., Porteous D.J., Deary I.J., Marioni R.E. (2017). Genetic prediction of male pattern baldness. PLoS Genet..

[83] Pirastu N., Joshi P.K., de Vries P.S., Cornelis M.C., McKeigue P.M., Keum N., Franceschini N., Colombo M., Giovannucci E.L., Spiliopoulou A. (2017). GWAS for male-pattern baldness identifies 71 susceptibility loci explaining 38% of the risk. Nat. Commun..

